# Technical Considerations in Ex Vivo Human Regulatory T Cell Migration and Suppression Assays

**DOI:** 10.3390/cells9020487

**Published:** 2020-02-20

**Authors:** Ahmad Adebayo Irekeola, Engku Nur Syafirah E. A. R., Norhafiza Mat Lazim, Rohimah Mohamud, Chan Yean Yean, Rafidah Hanim Shueb

**Affiliations:** 1 Department of Medical Microbiology and Parasitology, School of Medical Sciences, Universiti Sains Malaysia, Health Campus, Kubang Kerian 16150, Kelantan, Malaysia; profahmad007@yahoo.com (A.A.I.); engkunursyafirah@gmail.com (E.N.S.E.A.R.); yeancyn@yahoo.com (C.Y.Y.); 2 Department of Immunology, School of Medical Sciences, Universiti Sains Malaysia, Health Campus, Kubang Kerian 16150, Kelantan, Malaysia; rohimahm@usm.my; 3 Department of Otorhinolaryngology-Head and Neck Surgery, School of Medical Sciences, Universiti Sains Malaysia, Health Campus, Kubang Kerian 16150, Kelantan, Malaysia; norhafiza@usm.my; 4 Microbiology Unit, Department of Biological Sciences, College of Natural and Applied Sciences, Summit University Offa, Offa PMB 4412, Kwara State, Nigeria

**Keywords:** regulatory T cells (Tregs), suppression, migration, optimization

## Abstract

Regulatory T cells (Tregs) are renowned for maintaining homeostasis and self-tolerance through their ability to suppress immune responses. For over two decades, Tregs have been the subject of intensive research. The immunosuppressive and migratory potentials of Tregs have been exploited, especially in the areas of cancer, autoimmunity and vaccine development, and many assay protocols have since been developed. However, variations in assay conditions in different studies, as well as covert experimental factors, pose a great challenge to the reproducibility of results. Here, we focus on human Tregs derived from clinical samples and highlighted caveats that should be heeded when conducting Tregs suppression and migration assays. We particularly delineated how factors such as sample processing, choice of reagents and equipment, optimization and other experimental conditions could introduce bias into the assay, and we subsequently proffer recommendations to enhance reliability and reproducibility of results. It is hoped that prioritizing these factors will reduce the tendencies of generating false and misleading results, and thus, help improve our understanding and interpretation of Tregs functional studies.

## 1. Introduction

Regulatory T cells (Tregs) are a specific subset of CD4 T cells endowed with the ability to suppress immune responses, thus maintaining homeostasis and self-tolerance [1]. When naïve CD4^+^ T cells are triggered through their T cell receptors (TCRs) in the presence of appropriate cytokines, they differentiate into Th1, Th2, and Th17 effector T cells or Tregs [2]. Natural Tregs (nTregs), which develop in the thymus, and adaptive or induced Tregs (iTregs) produced from naïve T cells in the periphery, constitute the broad representatives of Tregs in the body [3]. Around 5%–10% of the peripheral naïve CD4^+^ T lymphocyte population in humans and mice are nTregs [3]. Although differences in the anatomical origins of these Tregs subsets are thought to influence their functional specificity [1], the intracellular Forkhead box protein 3 (FoxP3) is considered the most specific marker for all Tregs. Other surface markers like CD25, CD127 and TNFR2 are also used, in addition to FoxP3, to phenotype Tregs [4,5]. Due to their immunosuppressive ability, Tregs have been the subject of intensive research in the past few decades, especially in the areas of cancer, autoimmunity and vaccine development.

**Figure 1:**
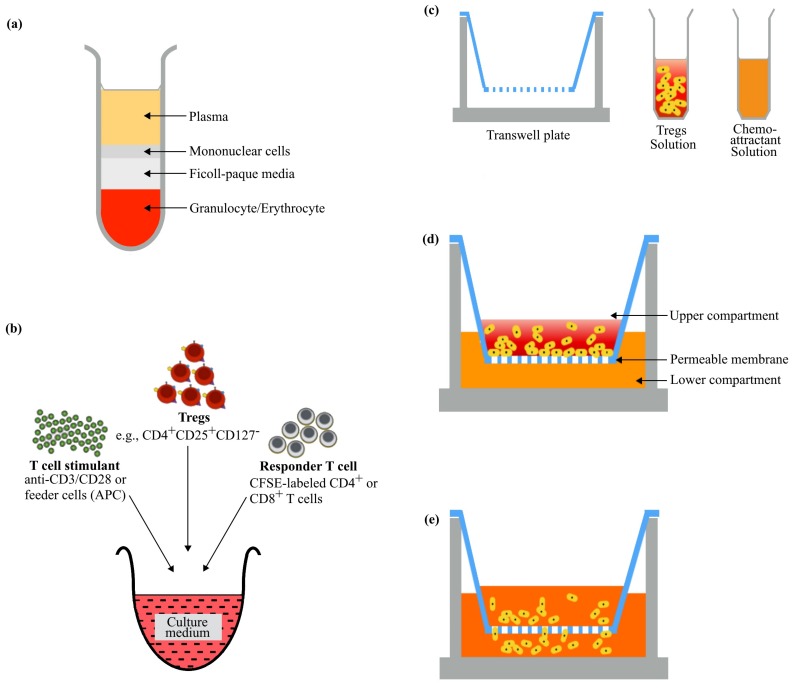
Basic Tregs suppression and transwell migration assay setup. (**a**) Schematic representation of distinct peripheral blood mononuclear cells (PBMC) layer following Ficoll density gradient centrifugation of whole blood. Tregs can be easily enriched from isolated PBMC through Magnetic-activated cell sorting (MACS) or Fluorescence-activated cell sorting (FACS) (**b**) Tregs suppression assay components. Suppression of the proliferation of responder T cells or repression of cytokine production is commonly assessed after 72 hours incubation. APC: Antigen presenting cells. (**c**) Tregs transwell migration assay components. (**d**) Assay setup prior chemotaxis. (**e**) Assay setup after chemotaxis. During incubation, Tregs move from upper compartment (membrane insert) to the lower compartment (receiver well) in response to signals from chemoattractant (e.g., CXCL12 and CCL22). Migrated cells can be enumerated using hemocytometer, flow cytometer or other dye assays.

The immunosuppressive potential of Tregs is commonly assessed in the Tregs suppression assay, a method that measures the suppression of responder cells (e.g., effector T cells) by Tregs in controlled conditions in vitro (Figure 1b). The suppression of proliferation of the responder cells could manifest as late or reduced proliferation or an absolute impedance of cell division. Suppression is also determined by evaluating the ability of Tregs to repress cytokine production by the responder cells [6]. For example, in cancer, interferon gamma (IFN-γ), one of the two main anti-tumor effector cytokines produced by activated CD8^+^ T cells, is suppressed by tumor necrosis factor receptor 2 positive (TNFR2^+^) Tregs [5]. Migration assay, on the other hand, is a technique used to assess the mobility of cells. Tregs migration assay relies on the principle of chemotaxis, the directional movement of cells towards a chemical gradient often established by signaling proteins (e.g., chemokines). Tregs are present in blood, tissues and the lymphatics and could inter-travel (e.g., from blood or tissue into afferent lymphatics) [7]. The movement of Tregs in steady state and during active immune responses in order to establish an adequate pool for effective function is often investigated using migration assay. In Tregs migration assay, the ability of Tregs to move toward a chemoattractant gradient is largely evaluated using a bare transwell insert and simply referred to as transwell migration assay. During the assay, Tregs are placed in a transwell containing a permeable membrane and inserted into a receiving well seeded with solution of test chemoattractant (Figure 1c–e). The setup is incubated, and the cells that migrate via the membrane to the receiving plate are subsequently enumerated. However, to assess Tregs migration through the endothelium, the transwell insert is layered with a monolayer of endothelial cells prior to treatment with Tregs. This type of migration assay is often termed transmigration or transendothelial migration (TEM) assay.

With the current global race to develop reliable immunotherapies against major diseases, Tregs migration and suppression assays are invaluable, since they are vital tools that aid deciphering the underlying functional roles of Tregs in autoimmune diseases, including multiple sclerosis [8,9]; type 1 diabetes [10,11]; rheumatoid arthritis [12,13] and cancers such as lung cancer [14], colorectal cancer [15], nasopharyngeal carcinoma [16] and breast cancer [17]. For example, to prevent allograft rejection, Tregs must migrate to both grafts and lymph nodes [18]. Furthermore, the migration and accumulation of functionally suppressive Tregs at tumor sites is associated with the advancement of cancer [19].

To perform either of these assays, Tregs and/or responder cells can be enriched from human or animal tissues or sourced from cell lines. While a number of researchers conduct Tregs functional studies in vivo, many others rely on ex vivo and/or in vitro approaches. Although ex vivo or in vitro studies may not fully depict the native cellular environment and conditions, they allow a fair simulation of the in vivo conditions, and more so, offer researchers relative ease, rapidity and even reduced ethical bottlenecks atypical of the in vivo approach. This review, however, focuses mainly on the ex vivo method as we concentrate on human Tregs isolation from blood and tissues and their subsequent use in functional studies.

## 2. Sample Processing and Preparation of Single-Cell Suspension

Given the low population of Tregs [20], sufficient samples should be collected when isolating human Tregs from peripheral blood mononuclear cells (PBMC) of whole blood or from tissue biopsy, particularly when immunophenotyping and when downstream experiments, such as Tregs migration and suppression assays, are to be conducted. This is vital, because the quantity of sample processed correlates directly with the number of cells that can be recovered. It should be noted that during the typical multiple-sample processing steps, including tissue digestion and immunostaining of single-cell suspension, some cells will die naturally or as a result of injury caused by mechanical disintegration and enzymatic digestion of tissues [21,22].

**Figure 2:**
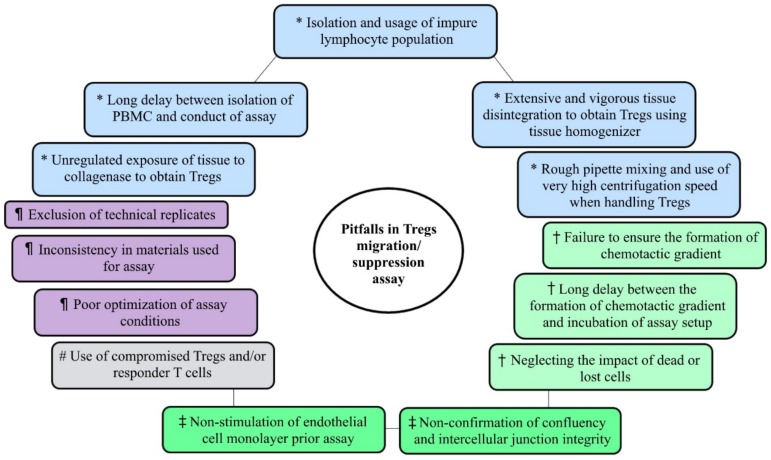
Some notable pitfalls in ex vivo human regulatory T cell migration and suppression assays. Pitfalls related to: *: sample processing to obtain Tregs, †: migration assay, ‡: transendothelial migration assay, #: suppression assay and ¶: both migration and suppression assay.

Even though isolation of PBMC from whole blood for Tregs functional studies appears to be less cumbersome, the protocol utilized should ensure a distinct layer of PBMC ensues following Ficoll gradient centrifugation (Figure 1a) to allow the isolation of the lymphocyte population with high purity. Interestingly, the purity of mononuclear cells can be enhanced by prior dilution of blood samples before Ficoll separation [23]. Additionally, it is important that the period between PBMC isolation and its usage for Tregs functional studies is reduced (Figure 2), as PBMCs begin to aggregate after sitting on ice for a long period of time. If the cell aggregates are not properly dissociated (it should be noted that some clumps can evade dissociation attempts), inaccurate enumeration of cells may arise.

Unless when immediate processing of tissue samples is not possible, the use of fresh tissue samples is recommended for the preparation of single-cell suspension. Slow-freezing, snap-freezing and overnight storage of intact tissues at 4°C have been shown to cause about 30%-90% reduction in total cell recovery [24].

Obtaining Tregs from tissue samples can be a little challenging especially, when trying to get rid of the extracellular matrix. Mechanical dissociation (e.g., the use of scalpel to mince into small pieces) and/or enzymatic digestion with collagenase is widely employed for the preliminary treatment of tissue prior to the derivation of single-cell suspension [25,26,27]. Vigorous tissue disintegration using tissue homogenizer rather than simple mechanical dissociation with scalpel, razor or scissors has been shown to be inappropriate for the preparation of single-cell suspension, as up to 50% and 27% decrease in cell yield and viability, respectively, characterized the usage of a homogenizer [22]. However, if the combination of mechanical dissociation and collagenase treatment is used, the concentration or incubation time of collagenase should be reduced, since the initial mechanical dissociation would increase the surface area of the tissue, allowing quicker digestion. Unregulated exposure of tissues to collagenase can affect the expression of certain molecules on the cell surface of leukocytes [28,29] (Figure 2). Similarly, it has also been suggested that enzymatic digestion could alter or damage the T cell receptor (TCR) on T cells [30]. Thus, these effects of collagenase might decrease recovery of the T cells of interest. Conversely, another study suggests only a minimal effect of collagenase on the intensity of cell surface markers and does not influence in vitro proliferation of T cells [31]. Hence, examining the potential effects of the collagenase utilized would enhance the reliability of results.

Controlling collagenase digestion is essential to sustaining Tregs viability at the sample preparation phase. Since collagenase digestion typically proceeds at an optimal temperature range of 35–37 °C or higher, the duration of digestion at the optimum temperature should be ascertained, as a longer incubation period could affect the survival of the cells. Further collagenase digestion can be impeded by continuing tissue processing at suboptimal enzyme conditions (e.g., temperature, enzyme concentration, etc.); repeated washing steps or the application of inhibitors such as EDTA and EGTA. Although it has been shown that different collagenase types (collagenases I, II, V and XI) exhibit similar activity on tissues [32], proper optimization should be conducted before processing actual samples. In addition, it is desirable to use only one type of collagenase for the digestion of all tissue samples under investigation to ensure consistency of method and, thus, reliability of results.

Following tissue digestion, the membranes of some cells inevitably rupture, releasing free DNA. As DNA are sticky in nature, they can initiate and facilitate cell aggregation in single-cell suspension, thereby compromising downstream assays. Addition of DNase I to tissue digestion cocktail would help mitigate this problem. Digested tissue samples are often strained using a cell strainer to get rid of tissue debris and clumps that arise in the course of tissue disintegration. The 70 µm strainer has been extensively used in the preparation of single-cell suspension for Tregs studies [33,34]. While sieving, a strainer should be changed once there is any suspected clog of the mesh’s pores to avoid loss of valuable cells. Alternatively, the sieve can be turned upside down and carefully flushed into a new tube followed by subsequent straining of the flushed matrix. It should be noted that straining of cells at low pressure is critical to minimizing stress on cells.

Other experimental procedures which may influence downstream results include centrifugation, vortexing and pipetting (Figure 2). Rough pipette mixing and vortexing can lead to premature rupture of cells, decreased cell viability and increased cell debris [22]. Similarly, subjecting cells to very high centrifugation speeds may damage cell membranes due to extreme compression of the cell pellet. On the other hand, when the centrifugation speed is too low, cells will fail to pellet and remain in suspension and might eventually be lost during washing steps. Centrifuging between 300 and 900 RCF would yield better results when processing tissue samples [22]. Overall, gentler handling of cells is necessary to maintain cell viability and higher cell recovery when preparing samples for further tests.

## 3. Optimization of Experiment

It is imperative to optimize every step and parameter of the Tregs migration and suppression assays as products from different manufacturers, as well as similar products but of different batches from the same manufacturer, may slightly differ in components. As factors such as equipment used, storage conditions of reagents, etc. can influence optimization results, we do not suggest specific optimum conditions here. It is, however, pertinent to report the optimum conditions used in each assay. For the migration assay, proper optimization should be done to particularly determine the ideal transwell to be used, volume and concentration of cells to be loaded into the transwell, volume and concentration of chemoattractant to be used in the receiver well, as well as incubation time. Assay conditions used in some recent Tregs studies are summarized and presented in Table 1. In the same vein, for the Tregs suppression assay, the optimum concentration and ratio of Tregs, responder cells and stimulants should be determined. As with actual experiments, both negative and positive controls should be included, and the assay should be done in at least three technical replicates during the optimization phase. In addition to correct optimization, highlighted in the next few paragraphs are areas we consider crucial to the success of a typical Tregs migration and suppression assay.

## 4. Tregs Migration Assay

### 4.1. Choice of Transwell

Generally, when conducting a migration assay, selecting an appropriate transwell is fundamental. Since transwells are manufactured with different pore sizes (e.g., 3 µm, 5 µm and 8 µm) to suit different experiments, care must be taken to avoid using a transwell whose pore size is too large or too small, as it will impact the outcome of the intended assay. A transwell whose pore size is too large will allow the free fall of cells from the upper compartment. Meanwhile, when the pore size is too small, cells are restrained from passage into the lower compartment of the assay setup. Results generated without due consideration of this factor could be misleading. Several studies have effectively used the 5 µm pore transwell in a migration assay [35,36,37]. Although Tregs may be larger than 5 µm, they can migrate via the pore since blood cells can alter their shape by rearranging the protein components of their cytoskeleton following the disruption of bonds holding the proteins together [38]. This property enables the cells to stretch and maneuver through the tiny pore. However, caution must be taken when interpreting results from studies that use atypical transwell pore sizes (e.g., 0.4 µm) [39] to investigate Tregs migration, since smaller pore sizes (0.4–3 µm) are mainly employed in studies of the transport of small chemical compounds [40]. Another crucial point to note when choosing a transwell for a Tregs migration assay is the volume of cell solution to be loaded into the transwell. Expectedly, this can vary depending on the size of the transwell; while a 24 mm transwell may be adequately seeded with 1000 µL of cell solution for optimum result, a 4 mm transwell would require much less volume. When too many cells are used, chances are that the pores of the transwell can become oversaturated. The utilization of fewer cells, on the other hand, could impair accurate enumeration of migrated cells in the receiver well. Additionally, when loading the transwell, contact of the pipette tip with the fragile transwell membrane must be completely eschewed to prevent inadvertent expansion of the transwell’s pore.

### 4.2. Endothelial Cell Monolayer

In the transendothelial migration (TEM) assay, confluency of cultured endothelial cells should be ascertained before proceeding with the assay. The use of stains such as silver nitrate [41], DAPI [42] and hematoxylin/eosin [18] as used in previous studies are helpful in confirming the formation and integrity of confluent monolayers. Further, intercellular junction integrity should be accounted for prior to the actual assay. This is pertinent, because Tregs infiltration most frequently occurs via endothelial junctions [43]. Thus, defective cell-cell junctions would inevitably result in an overestimation of migrated cells during the assay. Previous studies have employed immunohistochemical staining of the tight junction protein, occludin [44], and measurement of cell-cell junction permeability [41] to appraise the fitness of intercellular junctions.

TNF and IFN-γ stimulation has been demonstrated to aid leukocyte adhesion and TEM in different experimental models [45,46,47]. Stimulating endothelial cells during a transmigration assay would better simulate in vivo condition of Tregs migration. While information on endothelial cells stimulation with IFN-γ and/or TNF are discernible in reports from some Tregs TEM studies [35,48,49], it is obscured in other Tregs TEM studies [50,51]. This confounds the comparison of outcomes from the studies.

### 4.3. Establishing a Chemotactic Gradient

Given that chemotaxis of Tregs is largely dependent on ligand-receptor interactions [52,53], many studies today exploit the use of chemokine ligands to investigate the trafficking of Tregs to tumor microenvironments in various types of cancers [1]. This further underscores the significance of the careful isolation of Tregs when preparing and enriching the cells for migration assay. Damaged Tregs receptors stemming from incautious cell preparation steps can significantly skew the result of the migration assay, as distorted receptors would prevent the anticipated ligand-receptor interplay, leading to little or no chemotaxis. Since Tregs receptor integrity is not usually determined after isolation, it is pertinent to avoid harsh isolation protocols that might compromise cell surface receptors. Several chemokine ligands, including CCL22, CXCL12 and CCL28 [54,55,56,57], among others, have been used to investigate Tregs migration potentials in different types of cancers.

When the recipient well of the migration assay setup is seeded with solution containing the chemokines of interest, care must be taken to ensure the solution is in contact with the upper chamber bearing the Tregs to allow the formation of a chemotactic gradient. Once a gradient is formed, the setup should be gently handled to avoid spillage of contents from the top chamber to the lower one, as this can completely invalidate the results of the assay. Furthermore, incubation of the setup should be done immediately after the gradient is formed, because the transwell migration assay is only suitable for short-term investigation. After an extended period, the chemotactic gradient is lost since the concentration of chemokine ligands becomes equal in both chambers, consequently hampering the directional movements of Tregs from the top to the bottom well of the transwell setup.

### 4.4. Counting Migrated Cells

After incubation, the cells that migrate to the receiver well can be enumerated using a hemocytometer, flow cytometer or other dye assays (Table 1). However, it is important to note the quantity of starting Tregs, the migrated, as well as the non-migrated cells. This will help assess the impact of dead or lost cells on the results of the assay (Figure 2).

## 5. Tregs Suppression Assay

### 5.1. Choice of Responder T Cell

In Tregs functional studies, conventional T cells (Tconv) is a term often used to describe T cells that do not exhibit the classical phenotype of Tregs and are predominantly derived from CD4 and CD8 T cells. Unlike Tregs, Tconv play significant roles in pro-inflammatory responses and are considered effector T cells [2,66]. Tconv have been widely used as targets of Tregs suppression in several studies [35,65,67].

While the use of CD4 or CD8 Tconv as responder T cells in a Tregs suppression assay is researcher-dependent, allogeneic and autologous combinations of Tregs/Tconv must be carefully considered. Even though the use of autologous responder cells is presumed to be more physiologically suitable and avoids probable alloreactivity [33], they are not entirely without downsides. For instance, prior in vivo conditions, such as medications, the presence of other diseases, various metabolic and inflammatory factors and other unknown factors, may affect the replication of CD4^+^ and CD8^+^ T cells [33], leading to unsatisfactory results ex vivo. Secondly, autologous responder T cells isolated from patients who recently underwent immunosuppressive therapy will not be suitable for a Tregs suppression assay, as the responder cells may have been affected and be unable to divide irrespective of an ex vivo challenge with Tregs. Lastly, Tconv from patients with autoimmune diseases can develop resistance to Tregs suppression [6] as a result of induced cell-intrinsic changes [68]. Thus, to avoid misleading results, it is crucial to address factors capable of influencing the normal division of responder T cells.

Certain circumstances, like insufficient cell concentration and the need to debar bias from compromised responder T cells, might warrant the use of allogeneic responder cells from healthy individuals. When this option is resorted to, adequate standardization of the human Tregs suppression assay should be ensured, especially when Tregs functions from different patients are to be compared. It should be noted that responder T cells from different healthy donors may vary in their degree of response to stimulation and Tregs challenge [6].

### 5.2. Tregs/Tconv Ratio

Another important consideration in a Tregs suppression assay is getting the Tregs/Tconv ratio right. Tconv can become unresponsive to Tregs suppression when the ratio of Tregs to Tconv is tilted in favor of Tconv cells [68]. As much as possible, attempts should be made to determine the most suitable Tregs/Tconv ratio for every sample type. We do not recommend the use of a single Tregs/Tconv ratio when human Tregs suppression is investigated in different samples, as an ideal ratio for one sample may not necessarily reflect an appropriate ratio in another sample. For example, at fixed cell concentrations, the suppression outcome of a 1:1 Tregs/Tconv coculture when the Tregs are highly ”potent” will differ from a situation when they are “less potent”.

Other integral assay constituents should also be considered when determining the appropriate Tregs/Tcov ratio. For example, studies have shown that the use of soluble anti-CD3 stimulation or stimulation of T-cell receptors with lower concentrations of plate-bound anti-CD3 enhanced Tregs-mediated suppression of Tconv proliferation, as well as cytokine production, while robust TCR stimulation with plate-bound anti-CD3 favored the proliferation of Tconv in a coculture with Tregs [69,70]. In addition, co-stimulation with anti-CD28 enabled Tconv to resist Tregs suppression in a coculture [70,71,72]. Given that Tregs utilize different mechanisms in their functional roles [73,74,75], it is crucial to factor in the potential impact of stimulation conditions to the overall outcome of the assay. Lastly, to avoid bias from assay plates used to seed the selected ratios of Tregs/Tconv, the nature of the plate (U-, V- or F-bottom) should be carefully considered, and there should be consistency in usage, since the different plate types can influence the degree of cell interactions.

### 5.3. Monitoring Suppression of Proliferation

Thymidine incorporation and Carboxyfluorescein succinimidyl ester (CFSE) dilution are frequently used to appraise Tregs suppression of responder cells. It is crucial, however, to note the downsides associated with each of the methods. The use of ^3^H-thymidine incorporation depends on the premise that Tregs are anergic following in vitro stimulation with anti-CD3 monoclonal antibodies but responsive upon addition of IL-2, and they are therefore not expected to incorporate ^3^H-thymidine in the absence of IL-2 [76,77]. However, as with in vivo proliferation of Tregs, IL-2 can also be supplied ex vivo by Tconv in a coculture with Tregs, consequently contributing to Tregs division [74,78]. Thus, it is pertinent to note this proliferative potential of Tregs when employing ^3^H-thymidine incorporation in order to avoid underestimation of Tregs suppressive ability. Furthermore, including an analysis of cytokines produced by Tconv in the assay setup would help generate more significant data. Another drawback of thymidine incorporation is that the data of Tconv cellular division obtained is often generated towards the end of the whole assay, while it has been demonstrated that utmost suppression may occur within the starting hours of the interaction between Tregs and Tconv [67]. It would therefore be useful to add and pulse duplicate plates with ^3^H-thymidine at 24-h intervals for the entire duration of incubation.

While CFSE dilution helps to overcome some of the demerits of thymidine incorporation, difficulties with the resolution of CFSE peaks and the evaluation of results are a common challenge. The following points would be useful when employing CFSE: (a) To enhance the quality of CFSE peaks, doublets should be excluded from the gates, especially when cells are sourced from tissues. (b) In cases of poor CFSE peaks, it may be helpful to co-stain with Ki-67 in order to accurately locate the position of nondividing CFSE peaks of the responder cells. (c) CFSE-labeled cells are light-sensitive; thus, exposure to light can significantly compromise the final readout of the assay. (d) Lastly, proliferation of responder cells alone (i.e., in the absence of Tregs) should be at least 20% to avoid overestimation of Tregs suppression [79].

## 6. Concluding Remarks

Studies of Tregs functions are ever burgeoning, and the promise of Tregs as potential targets for immunotherapy, especially in cancer and autoimmunity, is continually being explored. To bolster the remarkable discoveries and progress on Tregs functions, the reliability and reproducibility of results is invaluable. We have discussed how assay materials and experimental conditions could impact the outcome of Tregs migration and suppression assays and the need to be wary of generating results that may be misinterpreted or misleading. We also summarized some of the notable pitfalls (Figure 2). Differences in the types of assay plates, transwell inserts, cell concentrations and ratios and method of results evaluation, among other factors, often makes the comparison of outcomes from different studies herculean. Thus, providing explicit assay protocols and stating clearly any assumptions made when conducting these assays would significantly ease the reproducibility of results and enhance the accurate interpretation of data.

## Figures and Tables

**Table 1:** Tregs migration assay conditions.

Transwell Type (T) & Pore Size (S)	Number (N) & Volume (V)^†^ of Cells in Transwell	Concentration (C) & Volume (V)^†^ of Chemoattractant in Receiver Well	Incubation Condition	Enumeration of Migrated Cells	Reference
T: 24-well S: 5 µm	N: 5 × 10^4^ V: NS	C: 100 ng/mLV: NS	37 °C, 4 h	Hemocytometer	[58]
T: 24-well* S: 5 µm*	N: 5 × 10^5^ *V: NS	C: 20 ng/mL V: NS	NS, 6 h	Flow cytometer	[34]
T: NS S: 3 µm	N: 1 × 10^6^ V: NS	C: NS V: NS	37 °C, 6 h	Flow cytometer	[59]
T: 96-well S: 5 µm	N: 1 × 10^5^ V: 100 µL	C: 500 ng/mL V: 150 µl	NS, 5 h	Flow cytometer	[60]
T: 24-well S: 5 µm	N: 1 × 10^6^ V: NS	C: 1 ng/mL V: 500 µL	37 °C, 4 h	Flow cytometer	[61]
T: 24-well S: 5 µm*	N: 2.5 × 10^5^ V: 100 µL	C: vary V: 600 µL	37 °C, 3 h	Flow cytometer	[62]
T: NS S: 5 µm	N: 3 × 10^5^ V: 100 µL	C: vary V: 600 µL	37^o^C, 4 h	Flow cytometer	[63]
T: 96-well S: NS	N: 1 × 10^5^ V: NS	C: 20 nM V: NS	37 °C, 4 h	Flow cytometer	[64]
T: NS S: 5 µm	N: 1 × 10^5^ V: 300 µl	C: 200 ng/mL V: NS	NS, Vary	Hemocytometer	[35]
T: 24-well S: 8 µm	N: NS V: NS	C: 100 ng/mL V: NS	37 °C, 3 h	Flow cytometer	[26]
T: 24-well S: 5 µm*	N: 2 × 10^5^ V: 100 µL*	C: vary V: 600 µL*	NS, 4 h	Hemocytometer	[65]
T: 96-well S: 5 µm	N: 1 × 10^5^ V: 50 µL*	C: vary* V: 150 µL*	37 °C, 90 min	Flow cytometer	[37]
T: 96-well S: NS	N: NS V: NS	C: 20 ng/mL V: NS	37 °C, 5% CO_2_ 4 h	Flow cytometer	[25]

NS: not stated in the publication, †: volume of cell and chemoattractant solution and *: data was obtained through direct correspondence with the author.
